# Mitochondrial Oxidative Stress Causes Hyperphosphorylation of Tau

**DOI:** 10.1371/journal.pone.0000536

**Published:** 2007-06-20

**Authors:** Simon Melov, Paul A. Adlard, Karl Morten, Felicity Johnson, Tamara R. Golden, Doug Hinerfeld, Birgit Schilling, Christine Mavros, Colin L. Masters, Irene Volitakis, Qiao-Xin Li, Katrina Laughton, Alan Hubbard, Robert A. Cherny, Brad Gibson, Ashley I. Bush

**Affiliations:** 1 Buck Institute for Age Research, Novato, California, United States of America; 2 Mental Health Research Institute of Victoria, and Department of Pathology, The University of Melbourne, Parkville, Victoria, Australia; 3 School of Public Health, EHS/Biostatistics, University of California Berkeley, Berkeley, California, United States of America; 4 Department of Psychiatry, Massachusetts General Hospital, Charlestown, Massachusetts, United States of America; Massachusetts General Hospital & Harvard Medical School, United States of America

## Abstract

Age-related neurodegenerative disease has been mechanistically linked with mitochondrial dysfunction via damage from reactive oxygen species produced within the cell. We determined whether increased mitochondrial oxidative stress could modulate or regulate two of the key neurochemical hallmarks of Alzheimer's disease (AD): tau phosphorylation, and ß-amyloid deposition. Mice lacking superoxide dismutase 2 (SOD2) die within the first week of life, and develop a complex heterogeneous phenotype arising from mitochondrial dysfunction and oxidative stress. Treatment of these mice with catalytic antioxidants increases their lifespan and rescues the peripheral phenotypes, while uncovering central nervous system pathology. We examined *sod2* null mice differentially treated with high and low doses of a catalytic antioxidant and observed striking elevations in the levels of tau phosphorylation (at Ser-396 and other phospho-epitopes of tau) in the low-dose antioxidant treated mice at AD-associated residues. This hyperphosphorylation of tau was prevented with an increased dose of the antioxidant, previously reported to be sufficient to prevent neuropathology. We then genetically combined a well-characterized mouse model of AD (Tg2576) with heterozygous *sod2* knockout mice to study the interactions between mitochondrial oxidative stress and cerebral Aß load. We found that mitochondrial SOD2 deficiency exacerbates amyloid burden and significantly reduces metal levels in the brain, while increasing levels of Ser-396 phosphorylated tau. These findings mechanistically link mitochondrial oxidative stress with the pathological features of AD.

## Introduction

Mitochondria are a main source of potentially pathogenic reactive oxygen species (ROS) such as superoxide, particularly in highly metabolically active organs such as the brain and heart [1]–[3]. The chief defense against superoxide produced during the course of respiration is mitochondrial superoxide dismutase (SOD2). Mice have been genetically modified to lack *sod2* in specific tissues [1], [4], or systemically in multiple genetic backgrounds [5], [6]. The resulting phenotypes include neonatal or embryonic lethality [5], [6], cardiomyopathy [5], [6], hemolytic anemia [7], [8], seizures [6], increased incidence of cancer [9], genomic instability [10], mitochondrial biochemical defects [11], [12], spongiform encephalopathy [3], optic neuropathy [4], movement disorders [3], [6] and neurodegeneration [12]. These phenotypes arise due to endogenous oxidative stress and several antioxidant interventions have been applied to the *sod2* nullizygous mouse model [2], [3], [13]. Some of these interventions have striking efficacy in preventing cardiomyopathy, extending lifespan, preventing spongiform encephalopathy [2], [3], reducing anemia [7], increasing mitochondrial enzyme activity, reducing neurodegeneration and attenuating gene expression characteristic of mitochondrially mediated spongiform encephalopathy [12], [14].

Alzheimer's disease (AD) is a progressive neurodegenerative disorder that is associated with oxidative stress [15] and the accumulation of two characteristic pathologies, neurofibrillary tangles, composed primarily of hyperphosphorylated aggregates of tau [16], and amyloid plaques, largely composed of aggregated Aß [17]. Aß binds copper and zinc selectively in AD tissue, and Aß:Cu complexes are a catalytic source of H_2_O_2_ [18], [19]. The molecular mechanism that results in an aggregation of Aß with increasing age in the brain is still uncertain, but abnormal interactions with biometals (principally copper and zinc) may play a prominent role [15]. To aid in the development of therapeutic strategies for AD, a number of animal models have been developed which recapitulate the plaque pathology [20]–[22]. However, only recently have transgenic mice been developed which model the two key neuropathologies associated with AD [23]. These mice progressively develop both amyloid and tau pathology, which are both associated with synaptic dysfunction. Of note in this genetic model, Aß deposition preceded the development of phosphorylated tau, and the distribution of phospho-tau closely recapitulated the distribution of tangles that characterize the human disease [24]. More recently, transgenic mice were developed which overexpressed mutant tau by 7–13 fold, resulting in mice that develop a tauopathy associated with neurodegeneration and impaired cognition [25]. Mice that model mitochondrial dysfunction and Alzheimers disease have only recently been developed [26], [27]. Mice overexpressing mutant APP were crossed with mice heterozygous for *sod2*, resulting in strains of mice with acceleration of amyloid accumulation, in addition to increasing behavioral defects in one strain [26].

A key question that remains unresolved in all currently available animal models of AD, is how well do they model AD where there are no mutations in either APP or tau? The majority of current animal models of AD are based on the overexpression of mutant forms of genes associated with AD linked to aggressive familial mutations [20], [23], [25], [28], [29]. Therefore, a significant need exists for the development of animal models of the neuropathology of AD, which are not based upon overexpression of mutant human proteins. Here we describe a novel model for AD neuropathology, showing that mice that lack *sod2* express AD-like hyperphosphorylation of tau, the protein component of neurofibrillary tangles. We show that this hyperphosphorylation of tau is attributable to mitochondrial oxidative stress. We also show that mice that are both transgenic for APP, and hemizygous for *sod2*, incur a higher level of tau phosphorylation, higher amyloid burden and decreased copper, zinc and manganese levels, consistent with a synergistic interaction between APP and mitochondrial oxidative stress in contributing to AD-like neocortical pathology.

## Methods

### Animal husbandry

*Sod2* nullizgyous mice of both sexes were genotyped between 2 and 3 days of age, and injected intraperitoneally with EUK189 at either 1 mg/kg (low dose) or 30 mg/kg (high dose) daily from 3 days of age until sacrifice, as previously described [2], [3]. EUK189 was purchased from Dalton Chemical Laboratories, Inc (Toronto, Canada). Wild type animals as well as *sod2* nullizygous mice were treated to control for compound specific effects. Mice were killed at 18–21 days of age and cortex harvested immediately for use in histological or biochemical studies.

Tg2576:*sod2* hybrid mice were created by crossing C57BL/6 *sod2* null mice (a kind gift of C.J. Epstein) to SJL mice to create F1 C57BL/6:SJL *sod2* heterozygotes. These mice were then crossed to APP mice (Tg2576 [20], on an identical genetic background), to generate Tg2576:*sod2* mice, which were maintained and bred as previously reported [20]. Tg2576:*sod2* mice, Tg2576 mice, and C57BL/6:SJL F1 hybrid *sod2* heterozygotes were maintained under barrier conditions until 497–527 days of age, when they were killed and neocortices were either harvested and frozen for biochemical analysis, or fixed in neutral buffered formalin for histological processing using standard protocols. We chose female Tg2576 mice to study because they have more robust plaque changes [30]. All animal procedures were carried out under approved IACUC animal protocols at the Buck Institute, which is AAALAC accredited.

### Immunohistochemistry

Parrafin embedded brain tissue was cut at a thickness of 8 µm, and tissue sections were de-paraffinised with xylene and re-hydrated through a stepwise series of ethanol solutions (100%, 95%, 70%). Non-specific background was reduced by a 5 minute pre-incubation in 0.5X PBS, in 10% methanol plus 3% hydrogen peroxide for 20 to 30 minutes. Antigen retrieval was routinely used with a commercially available kit (Antigen Unmasking Solution, Vector Lab., Burlingame CA). All slides were blocked (0.01X PBS, 5% v/v normal donkey serum, 0.1% Triton X-100) for 2 hours at room temperature, after which the primary antibody (rabbit anti-phospho-Ser 396 tau antibody, BioSource, Camarillo, CA) was added and left over 2 nights at 4°C. To ensure specificity of signal, a specific phospho-blocking peptide for phospho Ser-396 was used on adjacent serial sections (BioSource), which completely blocked the signal from the primary antibody. The secondary antibody (1∶500, Biotin-SP-conjugated donkey anti-rabbit IgG, Jackson ImmunoResearch Lab, West Grove, PA) was incubated for one hour at room temperature and the resulting signal was visualized using the Vectastain ABC kit and VectaDAB kit (Vector Laboratories, Burlingame, CA) according to the manufacturer's instructions. All slides were counterstained in Mayer's Haematoxylin for 30 seconds, and then dehydrated before the application of cover-slips, and subsequent photography using a digital camera (AxioVision v3.1, Carl Zeiss Vision). Silver staining for detection of possible neurofibrillary tangles, was performed as previously described [31], with the following modifications: immersion in the alkaline solution was 10 seconds, and the physical developer components for 25°C (10 mL solution A+5 mL solution B+5 mL solution C) were used immediately after mixing. Sections were developed in this solution until they had turned pale to mid-grey (approximately 20 to 30 minutes).

For evaluating amyloid load, we cut 7 µm coronal sections from formalin-fixed brain tissue. The sections were treated with 80% formic acid for antigen retrieval before probing with monoclonal antibody 1E8 that recognizes an epitope between residues 18 and 22 of Aβ (Glaxo SmithKline, UK). All sections were imaged on a Northern Light Illuminator (Imaging Research Inc, Ontario, Canada) using a Spot RT-KE 2MP digital camera (Diagnostic Instruments, MI, USA) equipped with a Nikkon 55 mm lens and 56 mm extension tube set. Each image was analyzed using ImagePro Plus 5.1 (Media Cybernetics, MD, USA). For each image an “area of interest” (AOI) line was drawn around the margins of the brain occupied by the hippocampus and cortex. The number of ß-amyloid-positive structures present in each section were then quantitated using color selection to separate the plaques from background labeling. A filter was also utilized to only include structures that were greater than 10 pixels in size. This data was expressed as the total number of ß-amyloid-positive pixels in each section and was exported to Microsoft Excel for analysis. Based upon the known size of each AOI, all values were normalized to allow comparisons between sections of different surface area and were expressed as pixels/mm^2^. Average Aß load was assessed in the cortex and underlying grey matter taken from two sections from each animal and all counts represent Aß found only in plaques. In all cases, the investigator undertaking the analyses was blinded to animal genotype.

### Western blotting and quantitation of phospho tau

**Figure 1:**
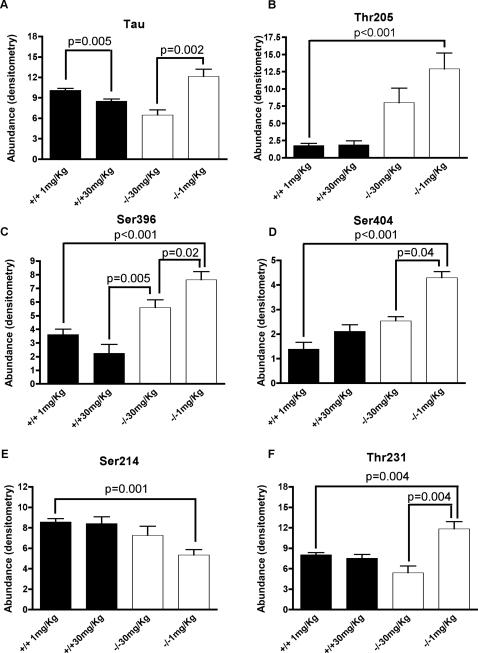
Mitochondrial oxidative stress causes hyperphosphorylation of tau. Western blots of cortical extracts from *sod2* null mice treated with 1 mg/kg (n = 8) of the catalytic antioxidant EUK189 or 30 mg/kg (n = 7) of EUK189 (white bars), or wild-type mice (n = 6, 1 mg/kg; n = 7, 30 mg/kg) treated comparably (black bars), were probed with the following antiphospho-tau antibodies; Thr 205, Ser 396, Ser 404, Ser 214, and Thr-231. Quantitation of chemiluminesence was carried out in the linear range of detection for each of these antibodies, using a regression model to adjust for the total amount of tau (results). P values for comparisons between each of the residues of phospho-tau were derived from the regression model, after correcting for the total amount of tau per group.

Protein was extracted from mouse cortex by homogenizing in tissue extraction buffer (50 mM Tris HCl (pH 7.4), 150 mM NaCl, 1 mM EDTA, 1% Triton, 0.1% SDS) containing mini complement protease inhibitors (Roche) at 4°C. Cellular debris was removed by centrifuging at 10,000 rpm in a bench top centrifuge for 5 minutes. Supernatant fractions were removed, and protein concentrations determined using a BCA protein assay kit (Pierce Biotechnology, Rockford, IL). Each sample was denatured in standard SDS/Page loading buffer and 2.5 µg of protein run on a NuPage 4–12% Bis Tris gels in MES buffer (Invitrogen, Carlsbad, CA). Proteins were transferred onto a Immuno-Blot PVDF membranes (Bio-Rad, Hercules, CA), according to the manufacturer's instructions. Blocking was carried in a standard 5% fat free milk solution in 1xTBS (0.05% Tween) for 1 hour at room temperature. Five anti-phospho-tau antibodies were employed which were directed against specific residues that had been previously identified as sites of hyper phosphorylation of tau in AD [32]; Thr-205, Ser-396, Ser-404, Ser-214, Thr-231 (Figure 1). Anti-phospho tau primary antibodies (Biosource, anti phospho-Thr 205, anti phospho-Ser 396, anti phospho-Ser 404, and anti phospho-Thr 214, all rabbit) (Chemicon, Temecula, CA) were typically used at a concentration of between 1∶1000 to 1∶10000, diluted in blocking buffer. The secondary antibody was an HRP-conjugated goat anti-rabbit (Pierce). Chemiluminescent detection was conducted with the ECL Plus western blotting detection system (Amersham Biosciences, Piscataway, NJ) and X-ray film according to manufacturer's instructions.

A number of preliminary blots were run for each antibody to determine the optimal conditions for quantification of phosphorylated tau. In these preliminary studies, the amount of protein was varied per gel, or the concentration of primary antibody varied in order to estimate the linear range of quantifiable phospho-tau.

Because the amount of tau protein in the sample could potentially contribute to the amount of phosphorylation at specific residues, we normalized by the total amount of tau in each sample. Instead of using a normalized outcome (such as the ratio of residue/tau), we adjust for the amount of tau using a simple linear modeling approach. Specifically, for each residue we estimate the model:
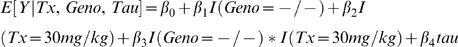
where *I(Geno = −/−)* is the indicator function ( = 1 if genotype is −/−, 0 otherwise, Tx is treatment). This model permits the testing and estimation of dose effects within genotype and similarly, genotypes within dose groups. The inference was derived using robust standard errors [33]. Note, in none of the models was the amount of total tau significantly associated with a specific outcome (i.e., the p-value of the test of *β_4_ = 0* was >0.05 for all residues). Comparison between specific groups using the above regression modeling after adjusting for total tau and their associated *p*-values are listed in Figure 1.

### Immunoprecipitation of phosphorylated tau

Immunoprecipitation was carried out using the Seize® Primary Immunoprecipitation Kit from Pierce, according to the manufacturer's instructions. 100 µg of Tau-5 anti-tau antibody (BioSource) was coupled to the 200 µl of AminoLink® Plus Coupling Gel Protein as per the manufacturer's instructions. Brain extracts (1.2 mg per mouse, for a total of 6 mg in 750 µl) were pooled from five 18-day-old *sod2* null mice that had been treated with a low dose of Euk189, or brain extracts (50 µl each) from nine female Tg2576:*sod2*+/− mice were pooled for a total of 1.8 mg in 450 µl. The pooled extracts were diluted to 5 ml with Binding Buffer, the 200 µl of antibody-coupled resin added, and the mixture rotated overnight at 4°C. Subsequent washes and elution were carried out as per the manufacturer's instructions. All bound tau protein was found to be eluted from the resin in the first 200 µl elution fraction.

### Proteolytic digestion of immunoprecipitated tau

Phosphorylated tau protein was either immunoprecipitated from single mutant (*sod2*−/−) mouse brain tissue as described above and was directly processed following a manual in-solution digestion protocol. Approximately 0.3–0.4 µg of immunoprecipitated tau in elution buffer (100 mM glycine) was brought to pH 8.0 by adding 50 mM NH_4_HCO_3_ buffer (final total volume 60 µl). Subsequently, acetonitrile (ACN) was added (40% final concentration) to denature the protein. The protein was incubated at 60°C for 30 min with a 500-fold molar excess of reducing reagent DTT over the number of cysteine residues in tau, followed by an incubation at 37°C for 45 min after adding a 1100-fold molar exess of alkylating reagent iodoacetamide (DTT and iodoacetamide were both purchased from Sigma, St. Louis, MO). After dilution of the reaction mixture to 10% ACN, the sample was incubated with 40 ng sequencing grade trypsin (Promega, Madison, WI) at 37°C for 8 hours. The resulting tryptic peptides were acidified with formic acid, and then subjected to mass spectrometric analysis.

### Mass Spectrometric Analysis (ESI-MS/MS)

The proteolytic peptide mixtures were analyzed by reverse-phase nano-HPLC-MS/MS. Briefly, peptides were separated on an Ultimate nanocapillary HPLC system equipped with a PepMap™ C18 nano-column (75 µm I.D. ×15 cm) (Dionex, Sunnyvale, CA) and CapTrap Micro guard column (0.5 µl bed volume, Michrom, Auburn, CA). Peptide mixtures were loaded onto the guard column and washed with the loading solvent (H_2_O/0.05% formic acid, flow rate: 20 µl/min) for 5 min, then transferred onto the analytical C18-nanocapillary HPLC column and eluted at a flow rate of 300 nl/min using the following gradient: 2% B (from 0–5 min), and 2–70% B (from 5–55 min). Solvent A consisted of 0.05% formic acid in 98% H_2_O/2% ACN and solvent B consisted of 0.05% formic acid in 98% ACN/2% H_2_O. The column eluant was directly coupled to a ‘QSTAR Pulsar i’ quadrupole orthogonal TOF mass spectrometer (MDS Sciex, Concorde, Canada) equipped with a Protana/ProXeon nanospray ion source (ProXeon Biosystems, Odense, Denmark). The nanospray needle voltage was typically 2300 V in the HPLC-MS mode. Mass spectra (ESI-MS) and tandem mass spectra (ESI-MS/MS) were recorded in positive-ion mode with a resolution of 12,000–15,000 FWHM. For collision induced dissociation tandem mass spectrometry, the mass window for precursor ion selection of the quadrupole mass analyzer was set to ±1 *m/z*. The precursor ions were fragmented in a collision cell using nitrogen as the collision gas. Spectra were calibrated in static nanospray mode using MS/MS fragment-ions of a renin peptide standard (His immonium-ion with *m/z* at 110.0713, and b_8_-ion with *m/z* at 1028.5312) providing a mass accuracy of ≤50 ppm. Samples were run at least twice by HPLC-MS/MS. Mass lists were generated of potential phospho-peptides that were observed as ESI-MS fingerprints but were not selected by ESI-MS/MS in the first HPLC run. These mass lists were entered as inclusion lists to the Information Dependent Acquisition of the QSTAR instrument (to be selected for MS/MS) in subsequent runs.

### Database searches for protein identification and additional bioinformatics programs

#### ESI-MS/MS data:

ESI-MS/MS data were submitted to our in-house search engine Mascot analyzing peptide sequence information from tandem mass spectra. The search engine Mascot uses a probability based ‘Mowse Score’ to evaluate data obtained from tandem mass spectra, e.g. for a score>37, protein matches are considered significant [34]. All acquired MS/MS spectra of phosphorylated peptides were interpreted and inspected “manually” and compared to the MS/MS spectra of their corresponding non-phosphorylated peptides.

#### ESI-MS data:

In order to obtain reliable peak lists from ESI-MS data that were generated during an LC-MS run, the programs Mascot Distiller (version 1.1.1) and Mascot Distiller MDRO Software Developers Kit (version 1.1.1) were purchased (Matrix Sciences, London, United Kingdom). An in-house developed java program named “MS-Assign” was used to interface with the Mascot Distiller software that was used for peak picking and to extract and compile ESI-MS peak lists from the LC-MS runs. Subsequently, ESI-PMF peak lists were submitted to our Mascot search engine or Protein Prospector.

### Evaluation of Aß load

We estimated Aß load using both immunohistochemistry (described above) and the DELFIA® Double Capture ELISA (Ritchie et al., 2003). Briefly, brain tissues (∼0.2 g) were homogenized in PBS at a ratio of 1∶10 (w/vol), and centrifuged at 100,000 g for 1 hr. The pellet was resuspended in the original volume and further solubilized with guanidine HCl to a final concentration of 5 M followed by centrifugation at 16,000 g for 20 minutes. To measure Aß40 or Aß42, the supernatants were diluted 1∶10 with blocking buffer (0.25% casein or Superblock (Pierce) in phosphate-buffered saline with 0.025% Tween-20) before adding to the plate. For the detection of Aß40 or Aß42 in the 100,000 g soluble fraction, 50 µl of the soluble fractions were applied to the ELISA plates. Plates were coated with either G210 (for Aβ40) or G211 (for Aβ42) antibodies, which are specific for the C-terminus of Aβ, then blocked with 0.5% (w/v) casein/PBS or Superblock/PBS buffer (pH 7.4). After washing the plates, WO2-Biotin was added to the wells. The epitope for WO2 is within residues 5–8. Aβ40 and Aβ42 peptide standards and samples were assayed in triplicate, and incubated overnight at 4°C. The plates were washed, europium labelled streptavidin added, and then developed with enhancement solution. Analysis was carried out using the Wallac Victor^2^ 1420 Multilabel Plate Reader (PerkinElmer, Melbourne, Australia) with excitation at 340 nm and emission at 613 nm.

Levels of the amyloid precursor protein (APP), the parent molecule of Aß, were quantitated by Western blot using the antibody 22C11 (Boehringer), which detects both the transgene-expressed human APP as well as the endogenous mouse APP. Briefly, snap-frozen tissues were thawed and homogenized in PBS (pH 7.4, 2 ml) and centrifuged at 100,000 g for 30 minutes. The pellet was resuspended 1∶1000 (w/v) in PBS and aliquots dissolved by extensive boiling in 8% SDS sample buffer containing 10% mercaptoethanol prior to separation on PAGE.

Statistical analysis was carried out between genotypes using a non-parametric *t*-test (two-sided) (PRISM, Graphpad, San Diego, CA).

### ICP-MS evaluation of metal content of soluble and insoluble amyloid fractions

Cortical samples were sonicated in 1 ml of PBS, centrifuged at 100,000 g for 30 minutes 4°C. The supernatant was collected and the pellet was resuspended in 1 ml of PBS.

A 500 µl aliquot of each brain pellet suspension was lyophilised. Samples were assayed by inductive-coupled plasma mass spectrometry according to our previously published procedures [35]. Results are expressed as the average of the three triplicates in micrograms of metal per g of protein (µg/g). Note: The levels of cobalt and aluminium in the samples were below detectable levels. Statistical analysis was carried out between genotypes using a non-parametric *t*-test (two-sided).

## Results

### Mitochondrial oxidative stress causes hyperphosphorylation of tau

To test whether mitochondrial oxidative stress causes hyperphosphorylation of tau, we studied *sod2* null mice that were treated with a catalytic antioxidant (EUK189). A low dose of EUK189 (1 mg/kg) rescues the peripheral phenotypes in these mice and extends the lifespan up to 3 weeks [2], while revealing a spongiform encephalopathy [14] and severe behavioral disorder [2]. This treatment response is concordant when *sod2* null mice are treated with other closely related antioxidant regimens (EUK8, EUK134, analogues of EUK189, [2]). In contrast, treating *sod2* null mice with a high dose of EUK189 (30 mg/kg) improves survival, behavioral abnormalities, and transcriptional responses associated with oxidative stress, in conjunction with increasing activities of mitochondrial enzymes that are sensitive to mitochondrial oxidative stress [2], [14], as well as preventing spongiform neuropathology. *Sod2* null mice that are not treated with antioxidants die within the first week of life from peripheral organ failure without any observable brain pathology [5], [11]. Hence they are unsuitable to examine the effects of endogenous oxidative stress on the brain.

By comparing the low and high dose antioxidant treated groups of mice, we were able to contrast the effects of mitochondrial oxidative stress on the potential phosphorylation status of tau. Mice were killed (N = 6–8 per treatment group), frontal cortices were harvested, and protein was extracted and western blotted for quantification of tau and phosphorylated tau. To control for drug specific effects, wild-type mice were also treated with either low or high dose of EUK189 for the same period of time. Untreated *sod2* null mice on this genetic background do not survive long enough (<1 week, [2]) to serve as a negative control.

First we asked whether lack of *sod2* caused an increase in the abundance of tau itself (Figure 1A). Two tailed non-parametric *t*-tests were carried out to determine differential levels of total tau in frontal cortices between *sod2* null mice and wild type controls. No significant differences were found between genotypes in either low (1 mg/kg) or high (30 mg/kg) EUK189 treated mice (Figure 1A). Next we asked if treatment with EUK189 within each genotype could have an effect on total tau levels. Interestingly, in wild type mice, there was a statistically significant drop in total tau (15%), as a result of increasing the concentration of EUK189 from 1 mg/kg to 30 mg/kg. In *sod2* null mice, the same trend was observed; increasing the dosage of EUK189 from 1 mg/kg to 30 mg/kg significantly decreased the total load of tau by 45%.

To determine whether lack of mitochondrial sod2 could alter the phosphorylation status of specific tau residues (Ser-396, Ser-404, Ser-214, Thr-231, and Thr-205), we normalized the amount of total tau per animal through a regression analysis (see methods). Thus, we examined the level of phosphorylated tau at each of these residues without being confounded by the differential loads of tau seen between groups in Figure 1A. Treatment with EUK189 has no effect on phospho-tau epitopes in wild type controls, however EUK189 normalized phospho-tau epitopes in the *sod2* mutant mice (Figure 1).

Figure 1B shows that in the low dose treated *sod2* null mice, there is a most prominent 7-fold increase in the hyperphosphorylation of tau at Thr-205 in *sod2* null compared to wild type mice (*p*<0.001). There is also a trend for reduced phosphorylation of Thr-205 due to a higher dose of antioxidant treatment, although this does not reach statistical significance (Figure 1B).

Figure 1C shows that lack of *sod2* causes a more than 2-fold significant increase in the level of hyperphosphorylation of Ser-396 (*p*<0.001) over treatment-matched controls, and that increasing the dose of antioxidant significantly attenuates the level of hyperphosphorylation (*p* = 0.02). Similarly, for Ser-404, mitochondrial oxidative stress also increases the level of hyperphosphorylation of tau to 3-fold over treated controls (Figure 1D, *p*<0.001), while increasing the dosage of antioxidant to reduce oxidative stress rescues the hyperphosphorylation to wild-type levels (*p*<0.04). Ser-214 is the only phospho-tau epitope that was hypo-phosphorylated in the low dose *sod2* null mice (*p*<0.001), with increased dose of antioxidant again normalizing the level of hypophosphorylation to wild-type levels (Figure 1E). Figure 1F shows an increase in the phosphorylation of Thr-231 (1.3 fold, p = 0.004) in the low-dose EUK-189 treated *sod2* null animals, which is correspondingly rescued with high-dose EUK-189 antioxidant treatment (p = 0.004). Finally we asked if there were any sex specific effects with regard to differential phospho-tau loads between treatment groups, and there were no significant trends detected (data not shown).

In order to validate our initial observations of hyperphosphorylation of tau at residues associated with AD in *sod2* null mice, we employed mass-spectrometry techniques as an alternate approach. To maximize the likelihood of detecting multiple sites of phosphorylation of tau, we immunoprecipitated total tau protein from *sod2* null (treated with 1 mg/kg EUK189) mice using the Tau-5 monoclonal antibody, which detects both phosphorylated and non-phosphorylated forms of tau (Biosource). After releasing tau from the antibody, the protein was reduced, alkylated and digested with trypsin (see methods). Peptides were analyzed by MALDI TOF MS for peptide mass fingerprint analysis, then nano-HPLC-ESI-MS/MS. To determine site(s) of phosphorylation within tau protein the ESI-MS/MS spectra were searched by Mascot, and spectra shown to contain phosphorylated peptides were verified by manual inspection. Our mass spectrometric results corresponded with the positive phospho-antibody probing of tau for phosphorylation sites shown in Figure 1, such as phospho Ser-404, phospho Thr-231, and phospho Ser-396 (as determined by ESI-MS/MS and ESI-MS). The tryptic peptides covering phosphorylation site phospho Thr-205 clearly showed mono- and diphosphorylation events, but due to the complex assembly of potential serine and threonine residues the mass spectrometric studies could not provide unambiguous assignment of Thr-205 as the phosphorylation site. Additional data seen in our mass-spec data provided clear evidence for the site of phosphorylation of phospho Thr-181 and phospho Ser-159, two well-characterized sites of phosphorylation of tau in AD [32]. Table 1 shows the range of sites identified by mass spectrometry in brain tissue isolated from *sod2* null mice.

**Table 1:** Tau phosphorylation sites identified in *sod2* null mouse brain tissue

residues	Detected by Antibody	% change a	mass spec ID b	phospho peptide observed by mass spectrometry	P site ID
Thr-205	Yes	728	+/−	c	MS c
Ser-404	Yes	309	+/−	S^396^PVVSGDT*pS^404^*PR^406^	MS/MS d
Ser-396	Yes	210	+/−	T^386^DHGAEIVYK*pS^396^*PVVSGDT*pS^404^*PR^406^	MS e
Ser-214	Yes	63	+	T^212^P**pS^214^**LPTPPTREPK^224^	MS/MS
Thr-231	Yes	147	+	V^226^AVVR**pT^231^**PPKSPSASK^240^	MS/MS
Ser-202	Yes	NS	+	S^195^GYSSPG**pS^202^**PGTPGSR^209^	MS/MS
Thr-181	No	−	+	T^175^TPSPK**pT^181^**PPGSGEPPK^190^	MS/MS f
Ser-159	ND	−	+	G^156^AA**pS^159^**PAQKGTSNATRIPAK^174^	MS/MS

**a**: Percent changes of listed residue in *sod2* null mice relative to *sod2*wild-types (normalized to 100%, see Figure 1), both treated with EUK189 at 1 mg/kg quantitated by Western blotting with monoclonal antibodies for the indicated tau residues, NS– not significant, ND – not done.

**b**: identifications were positive (+) if a MS/MS spectrum could be unambiguously interpreted to identify a unique phosphorylation site. In three cases (+/−) assignments were based on MS or MS/MS data and alternative sites were possible.

**c**: several matching phospho peptides observed by ESI-MS (i.e., peptides: mono-/di-phosph. S195-R209 and mono-/di-phosph. S181-R209);

**d**: Phosphorylate site possibly on Ser-404, Thr-403 or Ser-400;

**e**: peptide observed with 1P and 2P groups;

**f**: differences in peptide sequence (human vs. mouse) likely accounting for negative antibody result (homologous human peptide: TPPAPKTPPSSGEPPK);

**g**: no antibody available for Ser-159.

We next investigated whether pathological sequelae associated with hyperphosphorylation of tau in AD were also present in brain sections from *sod2* null mice. We used a modified Gallyas stain to detect possible neurofibrillary tangles (NFTs) in tissue sections (methods). We also used Schiff staining to determine if any other pathology related to AD, such as insoluble amyloid plaques were also present [36]. We were unable to identify any pathology relating to NFTs or aberrant amyloid metabolism using either of these methods in sections throughout the brain from 3-week-old *sod2* null mice on either low or high dose EUK189 (data not shown).

### Mitochondrial oxidative stress increases phosphorylation of tau in Tg2576:*sod2* mice

Having determined that mitochondrial oxidative stress causes hyperphosphorylation of tau, and that this can be ameliorated by antioxidant treatment, we next asked if there might be a synergistic interplay between aberrant amyloid metabolism and mitochondrial oxidative stress. Therefore, we crossed the Tg2576 (APP) mouse to hemizygous *sod2* mice to create Tg2576:*sod2* mice as described above. These double mutant mice are on an identical genetic background to Tg2576 mice. Therefore we aged four female genetically distinct groups (Tg2576, Tg2576:*sod2*, *sod2*+/−, and wild-type controls) on the same genetic background as the Tg2576 mice (F1 C57BL/6J: SJL hybrids), to approximately 16 months of age (when brain amyloid pathology is uniformly well-established in the Tg2576 model [20]) in order to investigate the effects of increased mitochondrial oxidative stress on amyloid and potential tau pathology. No difference in survival between the 4 groups was observed up to this age. The animals were sacrificed and the brains harvested, with half the brain being fixed and paraffin embedded for histological studies, while cortex from the other half was snap frozen for biochemical studies. Protein was extracted and equivalent amounts of protein from the 4 genetic groups were evaluated for levels of phospho Ser-396, a widely used epitope for detecting NFTs in AD, and readily induced in the brains of *sod2* null mice compared to controls (Figure 1).

**Figure 2:**
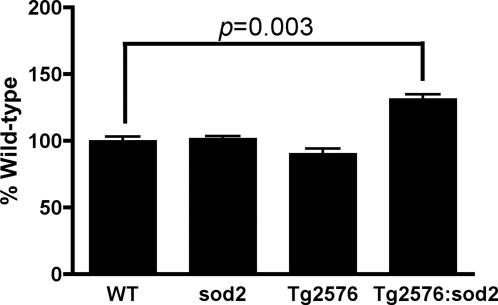
Mitochondrial oxidative stress results in a synergistic increase in the levels of phospho-tau in Tg2576 mice. Data show the mean±SD levels of Ser-396 phospho-tau assayed by Western blot of cortical extracts from mice of ≈500 days of age from each of the following genotypes: wild-type n = 4, *sod2*+/−n = 4, Tg2576 n = 8, Tg2576:*sod2* n = 9 (all matched for genetic background). A non parametric t test was carried out between genotypes, and a significant increase in phospho tau in the Tg2576:*sod2* mice was observed, compared to controls.

Levels of phospho Ser-396 in *sod2*+/− and Tg2576 mice were not significantly different from WT mice, indicating that oxidative stress induced by either loss of 50% *sod2* activity [9] or cerebral β-amyloid [19], was insufficient to impact levels of Ser-396 phosphorylation. However, we detected a significant 45% increase in the levels of phospho Ser-396 (*p*<0.003) in Tg2576:*sod2* over the levels in Tg2576 or *sod2*+/− alone (Figure 2). This is consistent with synergy between amyloid load and increased mitochondrial oxidative stress in the hyperphosphorylation of tau.

**Figure 3:**
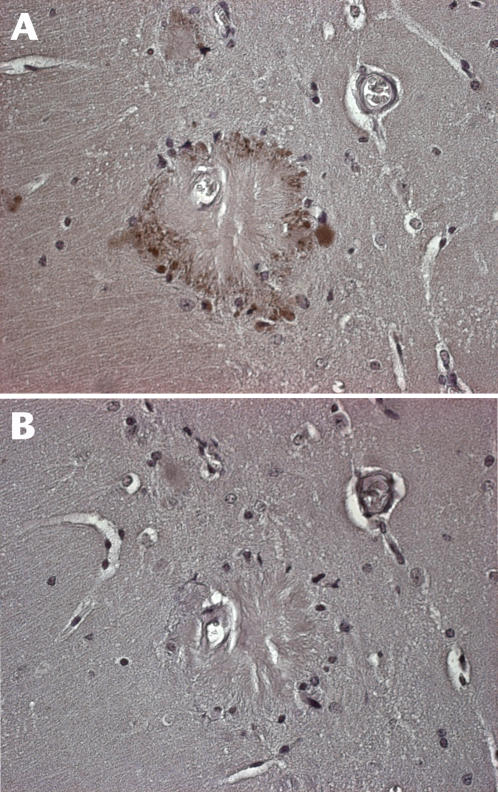
Immunohistochemical identification of phospho-tau positive dystrophic neurites in Tg2576:*sod2* mice. (a) Immunohistochemistry for anti Ser-396 was carried out as described in the text, and positivity was observed surrounding senile plaques in the brains of Tg2576:*sod2* mice. (b) In order to ensure specificity, we also tested the effects of preadsorbing the Ser-396 antibody with the peptide the antibody was raised against. This abolished the staining seen around the plaques.

Sections from fixed hemispheres of the same animals where Ser-396 phospho-tau levels were assayed in Figure 2 were stained for phospho Ser-396. In contrast to non-transgenic *sod2* null mice, we detected dystrophic neurites positive for phospho tau (Figure 3), in a pattern previously reported to be associated with plaque pathology [37]. Absorption with the peptide specific for the phospho Ser-396 epitope abolished detectable signal (Figure 3B.). Comparison of IHC for Ser-396 between Tg2576 and Tg2576:*sod2* mice (n = 3 each) revealed no qualitative or quantitative differences between the groups at the immunohistochemical level for hyperphosphorylated tau and no neurofibrillary tangles.

### Oxidative stress modulation is linked to Aß levels in Tg2576 mice

We next investigated the effect of *sod2* deficiency and Euk189 treatment upon brain Aß burden in APP transgenic mice.

**Figure 4:**
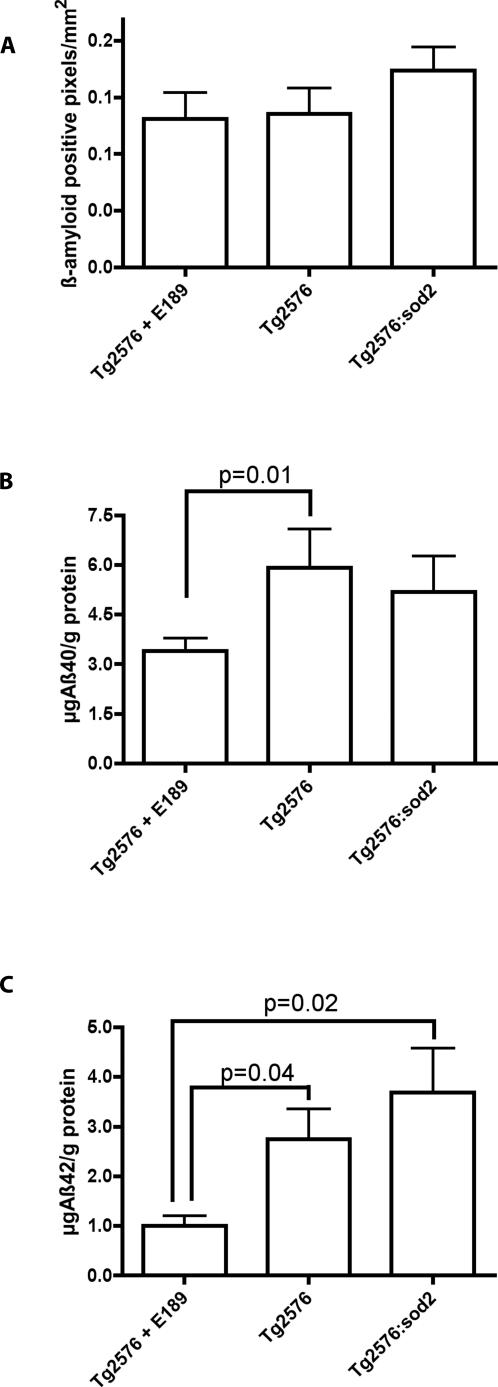
Exacerbation of amyloid load by genetic combination of lack of *sod2* and overexpression of APP. (a) Immunohistochemistry quantitation (area occupied by Aß/mm2) did not demonstrate any significant alterations in total Aß plaque burden, although there is an ∼30% increase (p = 0.2) in plaques in the Tg2576:*sod2* animals as compared to other groups. (b) ELISA quantitation demonstrates that EUK189 treatment significantly modulates Aß levels. Furthermore, genetically combining lack of *sod2* with overexpression of mutant APP elevates Aß levels in the Tg2576 mouse. The data indicate mean±SD.

We used two different methods to test this hypothesis; we evaluated Aß plaque load by immunohistochemistry and also assessed Aß protein by ELISA in each of the different treatment groups (Tg2576+EUK189, Tg2576, Tg2576:*sod2*). The immunohistochemistry data (Figure 4) indicate that the Tg2576:*sod2* animals have a 32% and a 29% increase in plaque burden as compared to Tg2576+EUK189 and Tg2576 animals respectively, which does not reach statistical significance (p = 0.18, p = 0.22). The ELISA data (Figure 4) demonstrates that Tg2576 and Tg2576:*sod2* animals have a 73% (p = 0.01) and 52% (p = 0.3) increase in total Aß1-40 and a 172% (p = 0.04) and 265% (p = 0.02) increase in total Aß1-42 respectively, as compared to Tg2576+EUK189 animals. Tg2576:*sod2* animals also show a 12% decrease in total Aß1-40 and a 34% increase in total Aß1-42 as compared to Tg2576 mice. These findings indicate that while the *sod2* mutant allele did not have a discernable effect on total Aß levels, the antioxidant treatment did drop Aß levels significantly.

**Figure 5:**
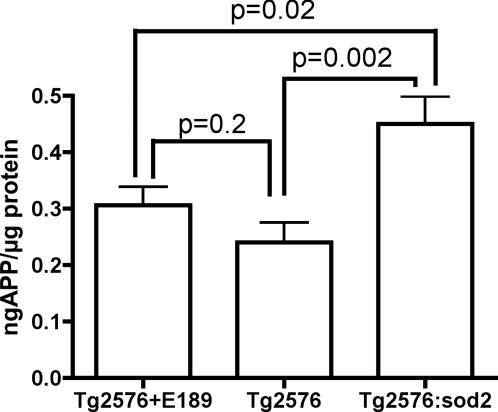
APP levels are elevated by genetic combination of *sod2* with Tg2576 mice. The data indicate mean±SD APP levels in brain assayed by western blot. As compared to the baseline Tg2576 levels, APP protein was marginally higher in Tg2576 mice treated with EUK189 (29% increased, p = 0.2), but markedly elevated by the presence of the sod2 mutation (88% increased, p = 0.002).

The precursor to Aß, the amyloid precursor protein (APP), also showed significant modulation according to genotype. As shown in Figure 5, the *sod2* mutation present in the Tg2576:*sod2* animals resulted in a significant elevation (87%, p = 0.002) in total APP levels as compared to the Tg2576 background strain and a 47% (p = 0.02) increase compared to Tg2576 mice treated with EUK189.

### Decreased metal content in Tg2576:*sod2* mice

Because APP overexpression lowers cellular copper [38], [39] and decreased copper levels exacerbates amyloid pathology [40], [41], we investigated whether the synergistic interplay between *sod2* ablation and Alzheimer pathology in Tg2576 transgenics is reflected by lowered brain metal levels.

**Figure 6:**
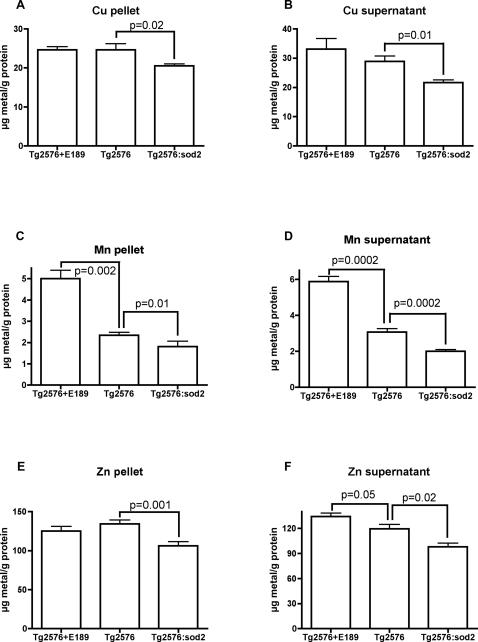
Metal levels are lowered by genetic combination of lack of *sod2* and overexpression of APP. Data indicate mean±SD brain metal levels in the various genotypes, as indicated. This revealed a significant decrease in insoluble (16%) (A) and soluble (25%) (B) Copper levels, insoluble (23%) (C) and soluble (35%) (D) Manganese levels, and insoluble (21%) (E) and soluble (18%) (F) Zinc levels in the Tg2576:*sod2* animals compared to the Tg2576 background strain. EUK189 treatment increased soluble (91%) and insoluble (113%) Manganese and soluble (12%) Zinc levels in the Tg2576 animals.

As shown in Figure 6, there is a decrease in copper levels in both the supernatant (25%, p = 0.01) and pellet (16%, p = 0.02) brain fractions of Tg2576:*sod2* animals as compared to the Tg2576 background strain. Similarly, there is a significant decrease in manganese levels in both the pellet (23%, p = 0.01) and supernatant (35%, p = 0.0002) fractions of Tg2576:*sod2* animals as compared to the background Tg2576 mice. There is also a significant increase in manganese levels in the EUK189 treated Tg2576 mice, as compared to the Tg2576 background strain, in both the pellet (113%, p = 0.002) and supernatant (91%, p = 0.0002) fractions. Similar trends are also shown for zinc, which shows a significant decrease in pellet (21%, p = 0.001) and supernatant (18%, p = 0.02) levels in the Tg2576:*sod2* animals as compared to the background Tg2576 mice. We also found a significant increase in supernatant zinc levels (12%, p = 0.05) in the EUK189-treated Tg2576 animals as compared to the background Tg2576 mice.

**Figure 7:**
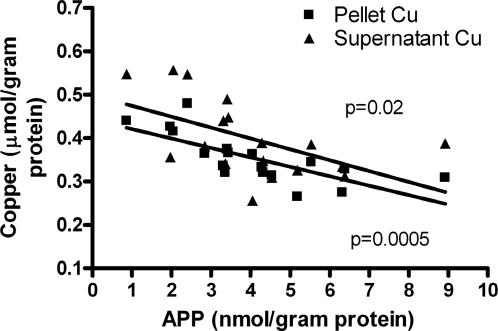
APP vs. copper correlation. Levels of APP and Copper in the soluble and pellet phases of brain homogenate from untreated Tg2576 and Tg2576:*sod2* crossed mice (pooled) were compared graphically and found to correlate strongly (* p<0.02).

Consistent with previous reports [38]–[41] and with an interaction of copper in our experimental system, we found that there is a significant inverse correlation between brain APP levels and both pellet (p = 0.0005) and supernatant (p = 0.02) copper levels (Figure 7) in the untreated transgenic mice. This implies that the decreased Cu levels in the APP:*sod2* double mutants (Figure 6A&B) are caused by elevated APP expression.

There was a marked decrease in the levels of Mn in both pellet and supernatant fractions of the Tg2576:*sod2* mice compared to the control Tg2576 mice. SOD2 is a homotetramer requiring Mn for its catalytic activity. Our observations of a 23–35% decrease in Mn in both the pellet and supernatant fractions from the brains of Tg2576:*sod2* mice compared to controls is consistent with the loss of SOD2. We also observed a 91–113% increase in the levels of Mn in the brains of mice treated with EUK189, which is a Mn-containing catalytic antioxidant. This is consistent with EUK189 or its bound Mn accumulating within the brain.

## Discussion

Mitochondria are the chief source of ROS within the cell, and age-related oxidative damage in the brain has been associated with neurodegenerative diseases such as Alzheimer's and Parkinson's diseases [15]. We tested the hypothesis that mitochondrial oxidative stress could contribute to the two pathological hallmarks of AD, amyloid burden and hyperphosphorylation of tau. We initially evaluated the brains of 3-week old *sod2* null animals treated with low and high doses of the catalytic antioxidant, EUK-189. These mice would die within the first week of life unless treated with antioxidants [2]. We previously developed the paradigm of treating *sod2* null mice with differential doses of catalytic antioxidants, whereby at a low dose of treatment (1 mg/kg of catalytic antioxidant), the peripheral phenotypes are rescued and lifespan is extended, but a severe spongiform pathology with neurodegeneration develops by 3-weeks-of-age [12]. At high doses of catalytic antioxidant (30 mg/kg), the spongiform pathology is completely prevented, and the accompanying neurodegeneration is attenuated [2], [12]. Thus, we could compare the changes in amyloid/tau in the brains of wild-type and *sod2*−/− mice treated with low and high doses of antioxidant (Figure 1) in order to test the hypothesis that oxidative stress from mitochondrial dysfunction promotes AD-like pathology.

We did not find evidence of increased amyloid burden in *sod2* nullizygous neonatal mice, possibly because endogenous mouse Aß has three amino acid substitutions that prevent mouse Aß from being precipitated by synaptic copper and zinc [42]. However, we did find a striking increase in the levels of hyperphosphorylated tau at 3 weeks of age, which responded to catalytic antioxidant therapy (Figure 1).

We observed a significant increase in the level of hyperphosphorylation in 4 out of 5 specific residues evaluated compared to control wild-type animals (Table 1). We did observe one residue (Ser-214) that was hypophosphorylated in response to mitochondrial oxidative stress. The phosphorylation status of various residues in tau results from a complex interplay between the inhibition and activation of kinases and phosphatases specific for individual residues. A recent report shows that a peptidyl-prolyl cis/trans isomerase (Pin1) caused hypophosphorylation of Thr-231, Thr-212, Ser-396, and Ser-404 in response to an extracellular hydrogen peroxide stress in primary neuronal cultures isolated from neonatal rats[43]. Hence, extracellular oxidative stress can cause hypophosphorylation of specific residues in tau. In contrast, we show here that intracellular oxidative stress appears to cause hyperphosphorylation in most residues we studied (table 1). Each of these hyperphosphorylated residues has been previously associated with aggregated hyperphosphorylated tau in AD [32], and in the high-dose treated *sod2* null animals, we observed a significant normalization of phosphorylation toward wild-type levels of phosphorylation in 3 out of 4 residues which were hyperphosphorylated (Figure 1). Thr-205 did not reach significance (although a trend consistent with the other three residues was apparent), possibly due to more noise in this particular group. Overall, we conclude that hyperphosphorylated tau which is caused by mitochondrial oxidative stress could be resolved with appropriate antioxidant therapy.

A number of prior studies have found associations between mitochondrial dysfunction and AD, and animal models of AD [26], [44]–[46]. Aß inhibits mitochondrial alcohol dehydrogenase [47], [48] and cytochrome oxidase [49] leading to increased mitochondrial oxidative stress and apoptosis [47]. A recent report also demonstrated a 9–11 fold increase in the expression of SOD2 by immunohistochemistry in the hippocampus of Alzheimer patients compared to controls, indicating a possible upregulation of antioxidant defenses in response to a pro-oxidant environment within the brain of AD patients [50]. To investigate the consequences of increased mitochondrial oxidative stress in the context of an established mouse model of AD, we crossed the Tg2576 mouse to mice heterozygous for *sod2*, a model of mild mitochondrial oxidative stress [9], [51], [52]. Concomitant with our observations of increased hyperphosphorylation of tau as a consequence of *sod2* knockout as described above (Figure 1), we investigated the levels of Ser-396 phospho-tau, which is characteristically elevated in AD [32], in the brains of double mutant mice compared to controls. We found a robust increase in the levels of phospho Ser-396 as a consequence of increased mitochondrial oxidative stress, above and beyond that seen in either parental control line, Tg2576 mice alone, or *sod2* heterozygotes (Figure 2). This implies that there is a synergistic interplay between mitochondrial dysfunction and amyloid burden that leads to hyperphosphorylation of tau. We also found a relationship between the level of oxidative stress and the level of amyloid burden (Figure 4). A recent report also found evidence of oxidative stress being linked to tau-mediated neurodegeneration in *drosophila* [53].

The trends we observed, of increased amyloid burden (Figure 4A) and Aß42 (Figure 4C) as a result of *sod2* heterozygosity, is consistent with a previous report where amyloid load was increased by approximately 2 fold compared to controls through genetic combination of *sod2* heterozygotes with the Tg19959 model of AD [27]. However, a more recent report observed decreased plaque burden in the parenchyma, with increased vascular amyloid, gliosis and exaggerated neurocognitive deficits as a consequence of *sod2* heterozygosity in J20 APP transgenic mice [26]. It is worth noting that Esposito et al., also found enhanced loss of microtubule-dependant protein 2 (MAP2) that was APP/Aß dependant in their *sod2*/APP model (which differs from the model we report here). MAP2 is involved in maintaining microtubule integrity like tau, and depletion of MAP2 is often observed in association with hypephosphorylation of tau[54]. Hence it is possible that the J20 APP/*sod2* mice[26], may also be experiencing hyperphosphorylation of tau, given the reported apparent loss of MAP2 in in the brains of these mice. Collectively, these three studies ([26], [27], and the data we report here) confirm that the SOD2 allele impacts upon AD-like pathology, but clearly the effects vary according to the AD mouse model utilized. Our study is the first to describe an association between SOD2 knockout or antioxidant-modulated oxidative stress and tau phosphorylation, which has not been investigated previously.

Previous studies have demonstrated that copper and iron levels in the brains of transgenic mice are lowered by expressing mutant APP or the carboxyl terminus of APP [39]–[41]. Consistent with metal deficiency fostering amyloid accumulation, we have previously found that amyloid clearance induced by clioquinol is associated with an elevation of cortical zinc and copper in the brains of Tg2576 mice [55]. Our current findings indicate that brain Cu, Zn, Fe, and Mn levels are all decreased as a consequence of genetic combination of Tg2576 and *sod2* heterozygotes. While the significance of this finding is not clear, these metals all participate in essential cellular function. It is likely that the drop in energy that results from the mitochondrial damage leads to a loss of brain metals. Decreased intracellular copper levels may exacerbate amyloid pathology [40], [41] by a mechanism that is not yet clear. Therefore, the drop in brain copper levels induced by the Tg2576:*sod2* double mutation may contribute to the increased amyloid burden in double mutants.

**Figure 8:**
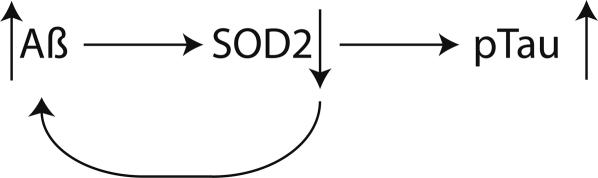
Model of potential interactions between increasing Aß, decreased activity of SOD2, and a resultant increase in the hyperphosphorylation of tau. As levels of Aß increase with age, this causes a concomitant decrease in the activity of SOD2, which in turn leads to increased aggregation of Aß. The resulting pro-oxidant state of the cell causes hyperphosphorylation of tau.

In summary, we have shown that mitochondrial oxidative stress per se causes hyperphosphorylation of tau, and that this can be prevented through appropriate antioxidant treatment (Figure 8). We have also demonstrated that the aggregate amyloid burden in a well established mouse model of AD (the Tg2576 mouse) is influenced by mitochondrial oxidative stress. This has implications for how we view mouse models of AD, in that to our knowledge, this is the first mouse model of AD that recapitulates two of the canonical hallmarks of AD pathology: plaques, and hyperphosphorylated tau, without resorting to overexpression of both mutant human proteins. Hyperphosphorylated tau is a component of neurofibrillary tangles, the formation of which is closely associated with the dementia process in AD. These findings encourage the use of ROS-scavenging antioxidants as being of potential therapeutic utility in AD.
